# [2-Amino-4,6-bis­(2-pyrid­yl)-1,3,5-tri­azine-κ^3^ *N* ^4^,*N* ^5^,*N* ^6^]dichloridocadmium(II)

**DOI:** 10.1107/S1600536811012517

**Published:** 2011-04-29

**Authors:** Man-Li Cao, Lei Shi

**Affiliations:** a Department of Chemistry, Guangdong University of Education, Guangzhou 510303, People’s Republic of China

## Abstract

In the title compound, [CdCl_2_(C_13_H_10_N_6_)], the 2-amino-4,6-bis(pyridin-2-yl)-1,3,5-triazine (HABPT) ligand adopts a tridentate tripyridyl coordination mode. The Cd^II^ atom is five-coordinated by three N atoms from the HABPT ligand and two chloride ions. In the crystal, mol­ecules are linked *via* N—H⋯N, N—H⋯Cl and C—H⋯Cl hydrogen bonds into a supra­molecular network.

## Related literature

For asymmetric ligands containing a triazine ring, see: Drew *et al.* (2000 ▶); Boubals *et al.* (2002 ▶); Medlycott *et al.* (2007 ▶); Chi *et al.* (2006 ▶); Cao *et al.* (2008 ▶, 2009 ▶). For the synthesis of the HABPT ligand, see: Case & Koft (1959 ▶). For metal complexes of the HABPT ligand, see: Drew *et al.* (2000 ▶); Boubals *et al.* (2002 ▶); Cao *et al.* (2009 ▶). For the diverse coordination modes of rigid multidentate polypyridyl ligands containing a triazine ring as a bridge, see: Zhou, Li, Wu & Zhang (2006 ▶); Zhou, Li, Zheng, Zhang & Wu (2006 ▶).
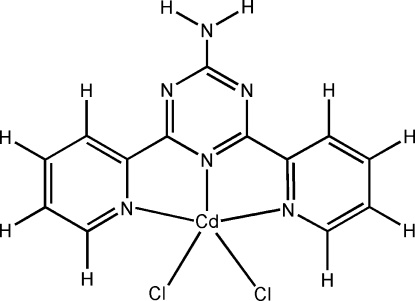


## Experimental

### 

#### Crystal data

[CdCl_2_(C_13_H_10_N_6_)]
*M* *_r_* = 433.58
Triclinic, 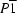

*a* = 8.8750 (6) Å
*b* = 9.2010 (7) Å
*c* = 10.2677 (7) Å
α = 82.5151 (9)°
β = 65.636 (1)°
γ = 82.798 (1)°
*V* = 754.89 (9) Å^3^
*Z* = 2
Mo *K*α radiation
μ = 1.80 mm^−1^
*T* = 293 K
0.34 × 0.31 × 0.28 mm

#### Data collection

Bruker SMART APEX CCD detector diffractometer
Absorption correction: multi-scan (*SADABS*; Bruker, 2005 ▶) *T*_min_ = 0.581, *T*_max_ = 0.636
5102 measured reflections
2588 independent reflections
2499 reflections with *I* > 2σ(*I*)
*R*_int_ = 0.014

#### Refinement

*R*[*F* ^2^ > 2σ(*F* ^2^)] = 0.020
*wR*(*F* ^2^) = 0.056
*S* = 1.01
2588 reflections
199 parameters
H-atom parameters constrained
Δρ_max_ = 0.28 e Å^−3^
Δρ_min_ = −0.30 e Å^−3^

### 

Data collection: *SMART* (Bruker, 2005 ▶); cell refinement: *SAINT* (Bruker, 2005 ▶); data reduction: *SAINT*; program(s) used to solve structure: *SHELXS97* (Sheldrick, 2008 ▶); program(s) used to refine structure: *SHELXL97* (Sheldrick, 2008 ▶); molecular graphics: *SHELXTL* (Sheldrick, 2008 ▶); software used to prepare material for publication: *SHELXTL*.

## Supplementary Material

Crystal structure: contains datablocks I, global. DOI: 10.1107/S1600536811012517/zq2094sup1.cif

Structure factors: contains datablocks I. DOI: 10.1107/S1600536811012517/zq2094Isup2.hkl

Additional supplementary materials:  crystallographic information; 3D view; checkCIF report

## Figures and Tables

**Table 1:** Hydrogen-bond geometry (Å, °)

*D*—H⋯*A*	*D*—H	H⋯*A*	*D*⋯*A*	*D*—H⋯*A*
N6—H6*A*⋯N3^i^	0.91	2.31	3.183 (3)	162
N6—H6*B*⋯Cl2^ii^	0.91	2.45	3.334 (2)	165
C12—H12*A*⋯Cl1^iii^	0.97	2.76	3.671 (2)	158
C2—H2*A*⋯Cl1^iv^	0.97	2.75	3.705 (3)	166
C11—H11*A*⋯Cl2^v^	0.97	2.82	3.596 (2)	138

Symmetry codes: (i) 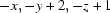; (ii) 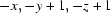; (iii) 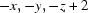; (iv) 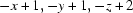; (v) 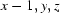.

**Fig. 1.**
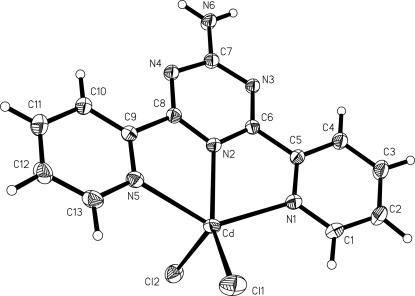
View of the title compound with the atom-labelling scheme. Displacement ellipsoids are drawn at the 30% probability level. H atoms are represented as small spheres of arbitrary radii.

## supplementary crystallographic information

### Comment

The rigid multidentate polypyridyl ligands containing a triazine ring as a bridge have attracted greatly our attention due to their coordination diversity (Zhou, Li, Wu & Zhang, 2006); Zhou, Li, Zheng, Zhang & Wu, 2006). Although coordination chemistry of the symmetrical ligands like tri(2-pyridyl)-l,3,5-triazine (TPT) has been well explored, the observations on the asymmetric ligands containing triazine ring are still rare (Drew *et al.*, 2000; Boubals *et al.*, 2002; Medlycott *et al.*, 2007; Chi *et al.*, 2006; Cao *et al.*, 2008, 2009).

The ligand 2-amino-4,6-bis(2-pyridyl)-l,3,5-triazine (HABPT) has five potential coordinate sites, it may offer a tridentate chelating mode or bis-bidentate binding mode with the capability of bridging two metal ions in chelating way (Drew *et al.* 2000; Boubals *et al.*, 2002; Cao *et al.*, 2009). As a contribution to the synthesis and structural studies of coordination abilities of the ligand (Case *et al.*, 1959; Drew *et al.*, 2000; Boubals *et al.*, 2002 and Cao *et al.*, 2009), we present here the crystal structure of the title compound, a new cadmium(II) complex with the HABPT ligand.

Within the title compound, C_13_H_10_CdCl_2_N_6_, the Cd^II^ center is five-coordinated respectively by three N atoms [Cd—N1 2.387 (2), Cd—N2 2.2679 (18), Cd—N5 2.433 (2) Å] from the HABPT ligand and two Cl atoms [Cd—Cl1 2.4176 (7) and Cd—Cl2 2.4431 (7) Å]. The ligand adopts a tridentate tripyridyl mode to coordinate to the Cd^II^ ion. Two chloride ligands are posited up and down the plane of the ligand that further accept hydrogen bonds from other ligands, the deviations values of Cd, Cl1 and Cl2 from the least-squares plane (rms deviation 0.122 Å for all non-H atoms of the planar tridentate ligand) are -0.603 (1), 0.540 (2) and -2.997 (1) Å, respectively. Viewed from the whole crystal structure, molecules are linked by intermolecular N—H···N, N—H···Cl and C—H···Cl hydrogen bonds to form a supramolecular structure. A weak intermolecular π–π interaction between the triazine ring and one pyridyl ring is also observed with a centroid-centroid distance of 3.976 (1) Å.

### Experimental

The ligand HABPT was prepared according to previously reported procedures (F.H. Case *et al.*, 1959). To a suspension of HABPT (0.025 g, 0.1 mmol) in 7 ml of ethanol was added the solution of CdCl_2_ (0.018 g, 0.1 mmol) in 7 ml distilled water. The resulting mixture was vibrated under ultrasonic condition for 20 min and then filtered. The obtained colorless filtrate was allowed to stay at ambient temperature for one week giving 0.026 g (60% yield, based on the ligand) of colourless crystals suitable for a structural determination.

Anal. Calcd. for C_13_H_10_Cl_2_N_6_Cd (%): C 36.01, H 2.31, N 19.38; found (%): C 36.12, H 2.40, N 19.31.

### Refinement

All H atoms were fixed geometrically and treated as riding with C—H = 0.96–0.99 Å and N—H = 0.90-0.91 Å with *U*_iso_(H) = 1.2 *U*_eq_(C or N).

### Crystal data

**Table:**

[CdCl_2_(C_13_H_10_N_6_)]	*Z* = 2
*M**_r_* = 433.58	*F*(000) = 424
Triclinic, *P*1	*D*_x_ = 1.908 Mg m^−^^3^
Hall symbol: -P 1	Mo *K*α radiation, λ = 0.71073 Å
*a* = 8.8750 (6) Å	Cell parameters from 2588 reflections
*b* = 9.2010 (7) Å	θ = 2.2–25.0°
*c* = 10.2677 (7) Å	µ = 1.80 mm^−^^1^
α = 82.5151 (9)°	*T* = 293 K
β = 65.636 (1)°	Block, colourless
γ = 82.798 (1)°	0.34 × 0.31 × 0.28 mm
*V* = 754.89 (9) Å^3^	

### Data collection

**Table:**

Bruker SMART APEX CCD detector diffractometer	2588 independent reflections
Radiation source: fine-focus sealed tube	2499 reflections with *I* > 2σ(*I*)
graphite	*R*_int_ = 0.014
φ and ω scans	θ_max_ = 25.0°, θ_min_ = 2.2°
Absorption correction: multi-scan (*SADABS*; Bruker, 2005)	*h* = −10→10
*T*_min_ = 0.581, *T*_max_ = 0.636	*k* = −10→10
5102 measured reflections	*l* = −12→12

### Refinement

**Table:**

Refinement on *F*^2^	Primary atom site location: structure-invariant direct methods
Least-squares matrix: full	Secondary atom site location: difference Fourier map
*R*[*F*^2^ > 2σ(*F*^2^)] = 0.020	Hydrogen site location: inferred from neighbouring sites
*wR*(*F*^2^) = 0.056	H-atom parameters constrained
*S* = 1.01	*w* = 1/[σ^2^(*F*_o_^2^) + (0.0354*P*)^2^ + 0.2818*P*] where *P* = (*F*_o_^2^ + 2*F*_c_^2^)/3
2588 reflections	(Δ/σ)_max_ = 0.001
199 parameters	Δρ_max_ = 0.28 e Å^−^^3^
0 restraints	Δρ_min_ = −0.30 e Å^−^^3^

### Special details

**Table:**

Geometry. All e.s.d.'s (except the e.s.d. in the dihedral angle between two l.s. planes) are estimated using the full covariance matrix. The cell e.s.d.'s are taken into account individually in the estimation of e.s.d.'s in distances, angles and torsion angles; correlations between e.s.d.'s in cell parameters are only used when they are defined by crystal symmetry. An approximate (isotropic) treatment of cell e.s.d.'s is used for estimating e.s.d.'s involving l.s. planes.
Refinement. Refinement of *F*^2^ against ALL reflections. The weighted *R*-factor *wR* and goodness of fit *S* are based on *F*^2^, conventional *R*-factors *R* are based on *F*, with *F* set to zero for negative *F*^2^. The threshold expression of *F*^2^ > σ(*F*^2^) is used only for calculating *R*-factors(gt) *etc*. and is not relevant to the choice of reflections for refinement. *R*-factors based on *F*^2^ are statistically about twice as large as those based on *F*, and *R*- factors based on ALL data will be even larger.

### Fractional atomic coordinates and isotropic or equivalent isotropic displacement parameters (Å^2^)

**Table:**

	*x*	*y*	*z*	*U*_iso_*/*U*_eq_	
Cd	0.21372 (2)	0.386656 (17)	0.788019 (17)	0.03420 (8)	
Cl1	0.22830 (10)	0.26193 (9)	1.00530 (7)	0.05660 (19)	
Cl2	0.41551 (8)	0.26525 (8)	0.58040 (7)	0.04683 (16)	
N1	0.2999 (2)	0.6218 (2)	0.7893 (2)	0.0354 (4)	
N2	0.0652 (2)	0.5692 (2)	0.7109 (2)	0.0316 (4)	
N3	0.0354 (2)	0.8157 (2)	0.6236 (2)	0.0307 (4)	
N4	−0.1444 (2)	0.6385 (2)	0.6264 (2)	0.0332 (4)	
N5	−0.0378 (2)	0.2992 (2)	0.7962 (2)	0.0352 (4)	
N6	−0.1636 (3)	0.8735 (2)	0.5305 (2)	0.0404 (5)	
H6A	−0.1322	0.9668	0.5070	0.048*	
H6B	−0.2468	0.8494	0.5095	0.048*	
C1	0.4108 (3)	0.6446 (3)	0.8398 (3)	0.0444 (6)	
H1A	0.4555	0.5567	0.8820	0.053*	
C2	0.4571 (3)	0.7822 (3)	0.8394 (3)	0.0472 (6)	
H2A	0.5370	0.7896	0.8800	0.057*	
C3	0.3859 (3)	0.9019 (3)	0.7847 (3)	0.0467 (6)	
H3A	0.4194	0.9989	0.7835	0.056*	
C4	0.2714 (3)	0.8806 (3)	0.7307 (3)	0.0377 (5)	
H4A	0.2264	0.9664	0.6864	0.045*	
C5	0.2318 (3)	0.7398 (3)	0.7343 (2)	0.0303 (5)	
C6	0.1044 (3)	0.7081 (2)	0.6848 (2)	0.0290 (5)	
C7	−0.0883 (3)	0.7746 (3)	0.5937 (2)	0.0321 (5)	
C8	−0.0629 (3)	0.5416 (2)	0.6838 (2)	0.0303 (5)	
C9	−0.1177 (3)	0.3903 (2)	0.7273 (2)	0.0311 (5)	
C10	−0.2455 (3)	0.3474 (3)	0.7005 (3)	0.0381 (5)	
H10A	−0.3033	0.4136	0.6498	0.046*	
C11	−0.2923 (3)	0.2053 (3)	0.7456 (3)	0.0481 (6)	
H11A	−0.3814	0.1696	0.7309	0.058*	
C12	−0.2127 (3)	0.1126 (3)	0.8178 (3)	0.0478 (6)	
H12A	−0.2415	0.0132	0.8551	0.057*	
C13	−0.0864 (3)	0.1634 (3)	0.8405 (3)	0.0427 (6)	
H13A	−0.0270	0.0998	0.8914	0.051*	

### Atomic displacement parameters (Å^2^)

**Table:**

	*U*^11^	*U*^22^	*U*^33^	*U*^12^	*U*^13^	*U*^23^
Cd	0.03892 (12)	0.02984 (12)	0.03786 (12)	0.00290 (8)	−0.02185 (9)	−0.00009 (8)
Cl1	0.0672 (4)	0.0623 (5)	0.0399 (3)	0.0064 (4)	−0.0279 (3)	0.0077 (3)
Cl2	0.0430 (3)	0.0568 (4)	0.0433 (3)	0.0013 (3)	−0.0188 (3)	−0.0137 (3)
N1	0.0387 (10)	0.0315 (11)	0.0427 (11)	0.0007 (8)	−0.0245 (9)	−0.0014 (8)
N2	0.0321 (10)	0.0272 (10)	0.0403 (10)	−0.0013 (8)	−0.0199 (8)	−0.0015 (8)
N3	0.0352 (10)	0.0268 (10)	0.0355 (10)	−0.0027 (8)	−0.0204 (8)	0.0005 (8)
N4	0.0390 (10)	0.0284 (10)	0.0389 (10)	−0.0042 (8)	−0.0236 (9)	0.0023 (8)
N5	0.0368 (10)	0.0294 (11)	0.0401 (11)	−0.0015 (8)	−0.0180 (9)	0.0021 (8)
N6	0.0485 (12)	0.0314 (11)	0.0551 (13)	−0.0068 (9)	−0.0372 (11)	0.0088 (9)
C1	0.0432 (14)	0.0467 (16)	0.0534 (16)	0.0046 (12)	−0.0312 (13)	−0.0062 (12)
C2	0.0435 (14)	0.0562 (18)	0.0551 (16)	−0.0070 (12)	−0.0313 (13)	−0.0081 (13)
C3	0.0494 (15)	0.0447 (16)	0.0565 (16)	−0.0134 (12)	−0.0295 (13)	−0.0030 (12)
C4	0.0429 (13)	0.0329 (13)	0.0436 (13)	−0.0066 (10)	−0.0237 (11)	0.0003 (10)
C5	0.0294 (11)	0.0331 (13)	0.0303 (11)	−0.0029 (9)	−0.0141 (9)	−0.0025 (9)
C6	0.0315 (11)	0.0275 (12)	0.0300 (11)	−0.0012 (9)	−0.0147 (9)	−0.0026 (9)
C7	0.0358 (12)	0.0307 (12)	0.0325 (11)	−0.0027 (9)	−0.0172 (10)	−0.0001 (9)
C8	0.0328 (11)	0.0278 (12)	0.0317 (11)	−0.0017 (9)	−0.0149 (9)	−0.0017 (9)
C9	0.0323 (11)	0.0279 (12)	0.0333 (11)	−0.0016 (9)	−0.0136 (9)	−0.0024 (9)
C10	0.0375 (12)	0.0340 (13)	0.0467 (14)	−0.0033 (10)	−0.0211 (11)	−0.0027 (10)
C11	0.0459 (15)	0.0414 (15)	0.0608 (17)	−0.0142 (12)	−0.0233 (13)	−0.0007 (13)
C12	0.0489 (15)	0.0310 (14)	0.0585 (17)	−0.0117 (11)	−0.0168 (13)	0.0048 (12)
C13	0.0440 (14)	0.0315 (13)	0.0474 (14)	−0.0008 (11)	−0.0166 (12)	0.0065 (11)

### Geometric parameters (Å, °)

**Table:**

Cd—N2	2.2679 (18)	C1—C2	1.378 (4)
Cd—N1	2.387 (2)	C1—H1A	0.9845
Cd—Cl1	2.4176 (7)	C2—C3	1.373 (4)
Cd—N5	2.433 (2)	C2—H2A	0.9723
Cd—Cl2	2.4431 (7)	C3—C4	1.386 (3)
N1—C1	1.336 (3)	C3—H3A	0.9727
N1—C5	1.349 (3)	C4—C5	1.376 (3)
N2—C6	1.331 (3)	C4—H4A	0.9838
N2—C8	1.337 (3)	C5—C6	1.489 (3)
N3—C6	1.324 (3)	C8—C9	1.485 (3)
N3—C7	1.362 (3)	C9—C10	1.384 (3)
N4—C8	1.311 (3)	C10—C11	1.385 (4)
N4—C7	1.356 (3)	C10—H10A	0.9818
N5—C13	1.333 (3)	C11—C12	1.375 (4)
N5—C9	1.348 (3)	C11—H11A	0.9664
N6—C7	1.321 (3)	C12—C13	1.381 (4)
N6—H6A	0.9078	C12—H12A	0.9670
N6—H6B	0.9080	C13—H13A	0.9815
			
N2—Cd—N1	68.97 (6)	C4—C3—H3A	122.2
N2—Cd—Cl1	141.58 (5)	C5—C4—C3	118.9 (2)
N1—Cd—Cl1	100.97 (5)	C5—C4—H4A	122.6
N2—Cd—N5	69.07 (7)	C3—C4—H4A	118.4
N1—Cd—N5	134.84 (7)	N1—C5—C4	122.3 (2)
Cl1—Cd—N5	101.44 (5)	N1—C5—C6	115.38 (19)
N2—Cd—Cl2	108.57 (5)	C4—C5—C6	122.3 (2)
N1—Cd—Cl2	109.67 (5)	N3—C6—N2	124.7 (2)
Cl1—Cd—Cl2	109.69 (3)	N3—C6—C5	120.0 (2)
N5—Cd—Cl2	98.80 (5)	N2—C6—C5	115.24 (19)
C1—N1—C5	117.9 (2)	N6—C7—N4	116.1 (2)
C1—N1—Cd	124.73 (17)	N6—C7—N3	118.9 (2)
C5—N1—Cd	117.40 (14)	N4—C7—N3	124.9 (2)
C6—N2—C8	116.34 (19)	N4—C8—N2	124.9 (2)
C6—N2—Cd	122.13 (14)	N4—C8—C9	118.8 (2)
C8—N2—Cd	121.48 (15)	N2—C8—C9	116.28 (19)
C6—N3—C7	114.17 (19)	N5—C9—C10	122.5 (2)
C8—N4—C7	114.62 (19)	N5—C9—C8	116.0 (2)
C13—N5—C9	117.9 (2)	C10—C9—C8	121.5 (2)
C13—N5—Cd	125.93 (16)	C9—C10—C11	118.7 (2)
C9—N5—Cd	115.21 (15)	C9—C10—H10A	122.5
C7—N6—H6A	120.1	C11—C10—H10A	118.8
C7—N6—H6B	120.9	C12—C11—C10	119.0 (2)
H6A—N6—H6B	119.0	C12—C11—H11A	118.6
N1—C1—C2	123.1 (2)	C10—C11—H11A	122.4
N1—C1—H1A	115.9	C11—C12—C13	118.9 (2)
C2—C1—H1A	121.0	C11—C12—H12A	123.4
C3—C2—C1	118.6 (2)	C13—C12—H12A	117.7
C3—C2—H2A	123.3	N5—C13—C12	123.0 (2)
C1—C2—H2A	118.0	N5—C13—H13A	116.2
C2—C3—C4	119.2 (2)	C12—C13—H13A	120.8
C2—C3—H3A	118.6		
			
N2—Cd—N1—C1	174.8 (2)	C7—N3—C6—N2	2.9 (3)
Cl1—Cd—N1—C1	33.5 (2)	C7—N3—C6—C5	−175.0 (2)
N5—Cd—N1—C1	152.02 (19)	C8—N2—C6—N3	−6.0 (3)
Cl2—Cd—N1—C1	−82.2 (2)	Cd—N2—C6—N3	171.45 (17)
N2—Cd—N1—C5	−5.55 (16)	C8—N2—C6—C5	172.00 (19)
Cl1—Cd—N1—C5	−146.86 (16)	Cd—N2—C6—C5	−10.6 (3)
N5—Cd—N1—C5	−28.3 (2)	N1—C5—C6—N3	−177.2 (2)
Cl2—Cd—N1—C5	97.44 (16)	C4—C5—C6—N3	5.9 (3)
N1—Cd—N2—C6	8.74 (16)	N1—C5—C6—N2	4.8 (3)
Cl1—Cd—N2—C6	89.73 (18)	C4—C5—C6—N2	−172.2 (2)
N5—Cd—N2—C6	171.65 (19)	C8—N4—C7—N6	177.5 (2)
Cl2—Cd—N2—C6	−95.81 (17)	C8—N4—C7—N3	−3.8 (3)
N1—Cd—N2—C8	−173.96 (19)	C6—N3—C7—N6	−179.0 (2)
Cl1—Cd—N2—C8	−92.97 (18)	C6—N3—C7—N4	2.3 (3)
N5—Cd—N2—C8	−11.05 (17)	C7—N4—C8—N2	0.3 (3)
Cl2—Cd—N2—C8	81.49 (17)	C7—N4—C8—C9	178.3 (2)
N2—Cd—N5—C13	−179.5 (2)	C6—N2—C8—N4	4.2 (3)
N1—Cd—N5—C13	−156.69 (19)	Cd—N2—C8—N4	−173.20 (17)
Cl1—Cd—N5—C13	−38.3 (2)	C6—N2—C8—C9	−173.76 (19)
Cl2—Cd—N5—C13	73.9 (2)	Cd—N2—C8—C9	8.8 (3)
N2—Cd—N5—C9	12.20 (16)	C13—N5—C9—C10	−0.6 (4)
N1—Cd—N5—C9	35.0 (2)	Cd—N5—C9—C10	168.70 (18)
Cl1—Cd—N5—C9	153.32 (16)	C13—N5—C9—C8	178.2 (2)
Cl2—Cd—N5—C9	−94.40 (16)	Cd—N5—C9—C8	−12.4 (3)
C5—N1—C1—C2	0.6 (4)	N4—C8—C9—N5	−174.8 (2)
Cd—N1—C1—C2	−179.7 (2)	N2—C8—C9—N5	3.3 (3)
N1—C1—C2—C3	0.1 (4)	N4—C8—C9—C10	4.0 (3)
C1—C2—C3—C4	−0.6 (4)	N2—C8—C9—C10	−177.8 (2)
C2—C3—C4—C5	0.4 (4)	N5—C9—C10—C11	−0.3 (4)
C1—N1—C5—C4	−0.9 (3)	C8—C9—C10—C11	−179.1 (2)
Cd—N1—C5—C4	179.46 (18)	C9—C10—C11—C12	1.2 (4)
C1—N1—C5—C6	−177.8 (2)	C10—C11—C12—C13	−1.2 (4)
Cd—N1—C5—C6	2.5 (3)	C9—N5—C13—C12	0.6 (4)
C3—C4—C5—N1	0.3 (4)	Cd—N5—C13—C12	−167.4 (2)
C3—C4—C5—C6	177.1 (2)	C11—C12—C13—N5	0.3 (4)

### Hydrogen-bond geometry (Å, °)

**Table:**

*D*—H···*A*	*D*—H	H···*A*	*D*···*A*	*D*—H···*A*
N6—H6A···N3^i^	0.91	2.31	3.183 (3)	162
N6—H6B···Cl2^ii^	0.91	2.45	3.334 (2)	165
C12—H12A···Cl1^iii^	0.97	2.76	3.671 (2)	158
C2—H2A···Cl1^iv^	0.97	2.75	3.705 (3)	166
C11—H11A···Cl2^v^	0.97	2.82	3.596 (2)	138

Symmetry codes: (i) −*x*, −*y*+2, −*z*+1; (ii) −*x*, −*y*+1, −*z*+1; (iii) −*x*, −*y*, −*z*+2; (iv) −*x*+1, −*y*+1, −*z*+2; (v) *x*−1, *y*, *z*.
